# Nanocarbons in Electrospun Polymeric Nanomats for Tissue Engineering: A Review

**DOI:** 10.3390/polym9020076

**Published:** 2017-02-21

**Authors:** Roberto Scaffaro, Andrea Maio, Francesco Lopresti, Luigi Botta

**Affiliations:** Department of Civil, Environmental, Aerospace, Materials Engineering, RU INSTM, University of Palermo, Viale delle Scienze, Ed. 6, 90128 Palermo, Italy; andrea.maio@unipa.it (A.M.); francesco.lopresti01@unipa.it (F.L.); luigi.botta@unipa.it (L.B.)

**Keywords:** graphene, CNTs, nanodiamonds, fullerene, biopolymer, tissue engineering, electrospinning, mechanical properties, electrical properties, antimicrobial properties

## Abstract

Electrospinning is a versatile process technology, exploited for the production of fibers with varying diameters, ranging from nano- to micro-scale, particularly useful for a wide range of applications. Among these, tissue engineering is particularly relevant to this technology since electrospun fibers offer topological structure features similar to the native extracellular matrix, thus providing an excellent environment for the growth of cells and tissues. Recently, nanocarbons have been emerging as promising fillers for biopolymeric nanofibrous scaffolds. In fact, they offer interesting physicochemical properties due to their small size, large surface area, high electrical conductivity and ability to interface/interact with the cells/tissues. Nevertheless, their biocompatibility is currently under debate and strictly correlated to their surface characteristics, in terms of chemical composition, hydrophilicity and roughness. Among the several nanofibrous scaffolds prepared by electrospinning, biopolymer/nanocarbons systems exhibit huge potential applications, since they combine the features of the matrix with those determined by the nanocarbons, such as conductivity and improved bioactivity. Furthermore, combining nanocarbons and electrospinning allows designing structures with engineered patterns at both nano- and microscale level. This article presents a comprehensive review of various types of electrospun polymer-nanocarbon currently used for tissue engineering applications. Furthermore, the differences among graphene, carbon nanotubes, nanodiamonds and fullerenes and their effect on the ultimate properties of the polymer-based nanofibrous scaffolds is elucidated and critically reviewed.

## 1. Introduction

Injury of organ and/or tissues has an important impact on quality of life and involves large social and economic costs. Using allogeneic grafts or other traditional treatments presents particular disadvantages such as risk of infection and immune rejection, as well as the limited availability of appropriate donor organs. Tissue engineering (TE) is an emerging area that gathers engineering and biological knowledge to create or restore injured tissues and organs by combining three basic tools: cells, biomaterials and biomolecules. 

In this context, TE requires scaffolds able to temporary replace the function of a living tissue from mechanical and physiological point of view exhibiting, at the same time, several physiochemical properties as well as biocompatibility [1,2,3].

Among the different approaches proposed for fabricating porous scaffolds for TE, electrospinning is one of the most investigated [4,5,6,7]. In fact, electrospinning is a versatile process technology exploited for the production of fibers with varying diameters, ranging from nano- to micro-scale. The electrospun nanofibers show high specific surface area, high porosity and tunable mechanical properties [7,8]. Thus, wide ranges of electrospun polymers have been extensively studied for application in catalysis [9], oil spill remediation [10], food packaging [11] and drug delivery [12]. TE pays particular attention on this technology since electrospun fibers offer topological structure features similar to the native extracellular matrix (ECM), thus providing an excellent environment for the growth of cells and tissues such as skin [13], bone [7,14], nerve [15] and vascular systems [16].

It is well known that, depending on the target tissue and/or on the type of disease, scaffolds for TE require specific features [2,7,17,18,19]. For this reason, in order to improve the physiochemical properties of biopolymeric electrospun structures, several scientific studies focused on the post-process surface modifications [20], blends with other biopolymers [21] and/or the use of nanofillers [7,22,23,24].

In particular, the scientific literature reports a wide number of nanoparticles (NPs) used to improve the bio/mechanical performance of nanofibrous mats such as biopolymeric NPs [24], nanoclays [25], saccharides nanocrystals [26], and nano-hydroxyapatite (HA) [27]. Among these, nanocarbons are now considered the most promising fillers for the development of high performance materials [28,29,30,31,32,33,34].

In fact, among the several fibers prepared by electrospinning, biopolymer/nanocarbons fibers exhibit huge potential applications combining the features of the biopolymeric matrix with the properties determined by the nanocarbons, i.e., conductivity and improved bioactivity. Furthermore, combining nanocarbons and electrospinning allows designing structures with nano- and microscale engineered patterns thus increasing the potential synergy between the matrix and the filler [7,35].

Many excellent and recent review articles have focused on the preparation and applications of electrospun fibers for tissue engineering have been published [5,36,37,38,39]. Most of them mainly summarized the exciting works emerging in a specific kind of polymer [40,41,42,43] or a specific TE application [4,44,45,46,47,48,49]. At the same time, several interesting reviews are focused on the preparation and characterization of nanocarbons used as filler for polymers [50,51,52,53,54,55,56,57]. However, to the best of our knowledge, no review papers deal with the synergistic effect of nanocarbons and polymeric electrospun fibers on the performance of nanofibrous scaffolds for tissue engineering. Moreover, the structure-property relationship for this novel class of systems is still far from being fully elucidated. This article aims to overview the most recent advances on electrospun polymeric nanomats containing nanocarbons for tissue engineering applications, paying particular attention to their structure-property relationship.

## 2. Electrospinning for Tissue Engineering

Electrospinning is a simple and versatile method to prepare ultra-thin fibers from polymer solutions or melts [58]. 

**Figure 1:**
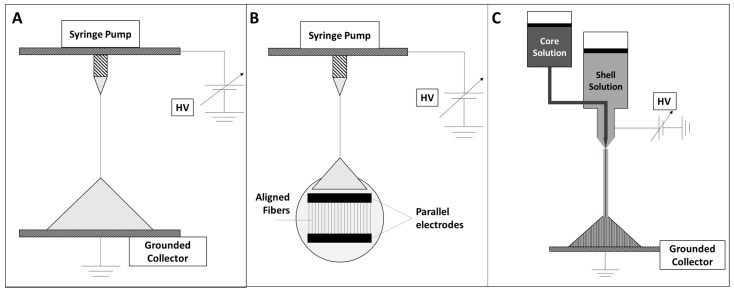
Schematic representations of (**A**) Conventional electrospinning setup; (**B**) parallel electrodes setup for aligned fibers; (**C**) coaxial electrospinning setup for core shell fibers.

In Figure 1A–C we schematically represented three different electrospinning setups. Conventional electrospinning (Figure 1A); Parallel electrodes setup for aligned fibers (Figure 1B) and coaxial electrospinning setup for core-shell structured fibers (Figure 1C).

The conventional electrospinning setup consists of three fundamental components: a high-voltage power source, a collector and a spinneret, as schematized in Figure 1A. The spinneret is usually connected to a syringe that is fed through the spinneret with a syringe pump. In front of the spinneret, at an appropriate distance, the collector is positioned so it can be either static or rotating. In order to convert the polymer solution to a charged polymer jet, a high-voltage/low-current power system is required (usually up to 30 kV) to charge the jet from the spinneret tip toward the surface of the fiber collector [59].

The electrospinning process can be controlled by several variables able to affect the fibers diameter and their surface topology, i.e., polymer molecular weight, applied voltage, solution flow rate, polymer concentration and electrode-collector distance [58,59]. 

Electrospinning is one of the most widely studied processing techniques used to produce porous biomaterials to be cultivated with cells and it has also been demonstrated as giving the most promising results in terms of TE applications [5]. In fact, electrospun scaffolds are able to mimicking the architecture of the ECM thus showing great advantages for TE. The ECM has a structure consisting of a 3D fiber network; it surrounds the cells in tissues and mechanically supports them. An ideal scaffold should mimic as much as possible the structure and function of the natural ECM, until the seeded cells have formed their own ECM [5]. 

Electrospinning allows the preparation of fibrous mats from a broad range of materials of natural and synthetic origin. Furthermore, the electrospun scaffolds show a total porosity up to 90% that is highly required in many TE applications [60]. 

Moreover, electrospinning is a versatile way to prepare scaffold mimicking functional gradients of living tissues. For example, layer-by-layer electrospinning permits to prepare scaffolds exhibiting gradients in composition, microstructure and porosity [19]. 

**Figure 2:**
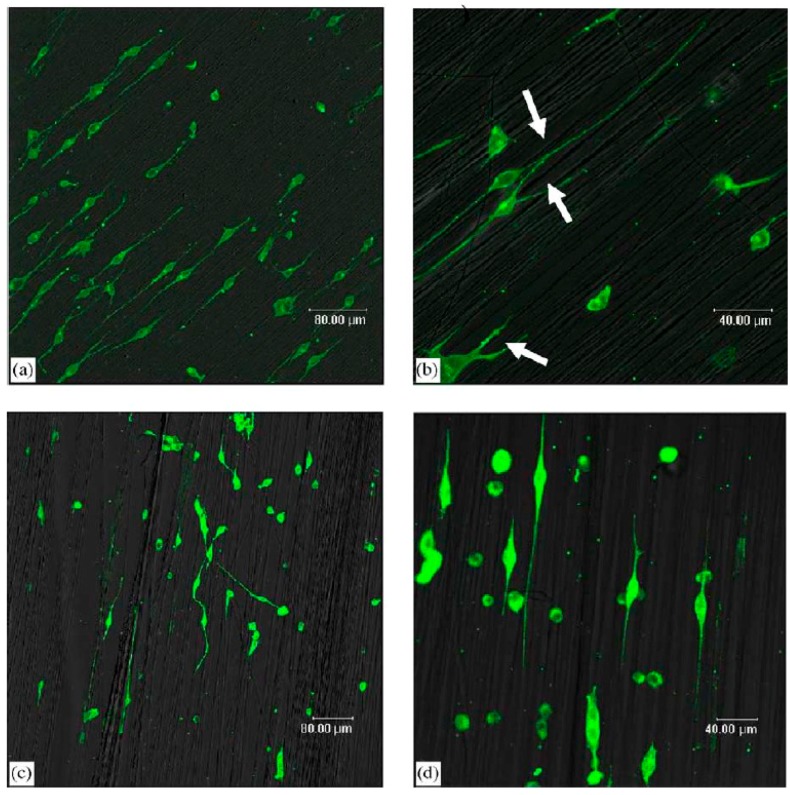
Laser scanning confocal microscopy micrographs of immunostained neurofilament 200 kD in neuronal stem cells after 2 days of culture; (**a**) on aligned nanofibers, low magnification (×200); (**b**) on aligned nanofibers, high magnification (×400); (**c**) on aligned microfibers; low magnification (×200) and (**d**) on aligned microfibers, high magnification (×400) Reprinted from [62] with permission from Elsevier.

For some TE applications, specific fiber arrangements could be more appropriate than conventional random mats. For instance, in nerve or muscle regeneration, the cells should grow along specific directions [61,62]. In these cases, the scaffolds may be required to display aligned fibers, parallel to each other, in order to guide the cell morphology, as shown in Figure 2. Yang et al. demonstrated that the neuronal stem cells elongated and their neurite outgrew along with the fiber direction for the aligned scaffolds, whereas the neurites were randomly orientated in scaffolds without fiber alignment [62].

The easiest way to obtain aligned fibrous structures exploits the presence of two parallel electrodes, able to break the axial symmetry of the deposition, thus causing the formation of parallel fibers, as shown in Figure 1B [63]. Otherwise, the fibers alignment may be achieved by using a cylindrical collector rotating at high speeds. 

Unconventional electrospinning setups are also required in such cases where electrospun mats are designed to carry specific functional materials for drug delivery, e.g., for biological activities, etc. [63,64,65,66,67,68]. In this context, the preparation of core-shell fibers may be requested. Among these, coaxial electrospinning can be conveniently adopted for the fabrication of core-shell fibers composed of different polymers, as well as hollow fibers [63,64,65,66,67,68]. This particular technical set-up, as clearly visible in Figure 1C, involves the presence of two concentric dies, respectively connected to two syringes containing different solutions. 

McKeon-Fischer et al. used this approach to prepare a core-shell structure constituted by an inner fiber made of a conductive polycaprolactone-carbon nanotubes nanocomposite and an outer sheath based on a biocompatible hydrogel. In this way, the authors were able to fabricate a self-contained actuating scaffold for skeletal muscle TE [35].

The choice of polymer matrix for electrospun TE scaffolds depends on the final application, the nature of the tissues to be regenerated and their regeneration time. In fact, each material exhibits specific mechanical properties, wettability, bioactivity and degradation rates [69].

Usually, polymers for TE applications are designed to match the regeneration rate of tissue in order to disappear when the cells begin to regenerate it. Biocompatible and biodegradable natural and synthetic polymers such as polyglycolides (PGA) [70], polylactides (PLA) [1,2,17,26,71], polycaprolactone (PCL) [7,17,18,72], various copolymers [43], polyurethanes (PU) [73], collagens [74,75], gelatin [76], chitosans [77], silk fibroin (SF) [41,73], and alginates [78] are extensively investigated for this purpose. NPs are likely able to affect these features of the polymer matrices thus giving the designer the possibility to tune specific properties or even endow the materials with additional features [7].

## 3. Nanocarbons for Tissue Engineering

The terms nanocarbons, nanostructured carbons and carbon-based nanomaterials are commonly used to indicate an extremely wide and variegated range of carbon materials possessing at least a tailored nanoscale dimension and physical-chemical features significantly affected by their nanoscale characteristics. Carbon nanotubes (CNTs) and graphene-based nanocarbons belong to this class of materials comprising even other forms of nanostructured materials, including fullerene, nanofibers, -diamonds, -horns, -onions, -coils and so on [79]. 

**Figure 3:**
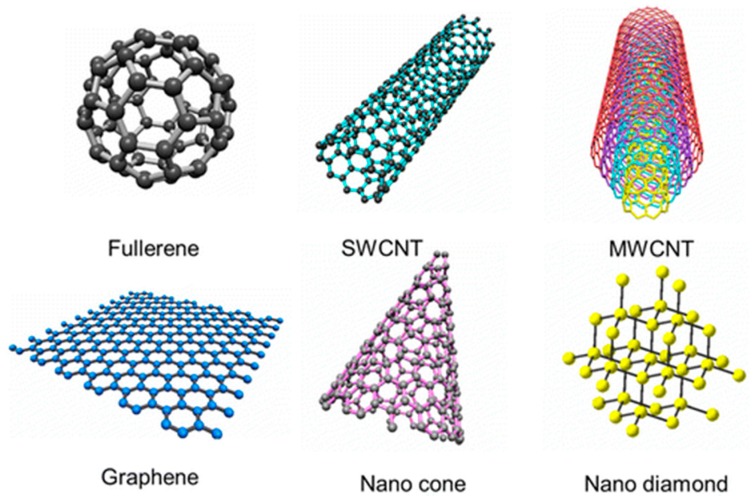
Schematic illustration of some nanocarbon. Reprinted with permission from [79]. Copyright (2013) American Chemical Society.

Figure 3 illustrates some forms of nanocarbons, each one being characterized by different geometry and degree of sp^2^ (or sp^3^) hybridization. These latter features can be used to categorize them into different classes and, on the other hand, can be adjusted or tuned in order to meet broad-spectrum requirements that enable them to be used in an extremely wide range of application fields. In fact, due to their unique structure and excellent mechanical, optical and electrical properties, briefly summarized in Table 1, as well as their outstanding lightness, nanocarbons are fast emerging as zero-, one- and two-dimensional wonder materials. Due to the possibility to achieve an extremely wide range of tailored properties upon varying their structure, nanocarbons are extensively studied in applications going from photonics and optoelectronics to biotechnology and nanomedicine, advanced electrodes, supercapacitors and polymer composites [80]. Indeed, emerging trends show that their exceptional properties can be exploited for biomedical applications, especially in drug delivery and TE [81]. In this context, nanocarbons offer intriguing physic-chemical and biological features for biomedical applications due to their nanometric size, large specific area and ability to interface/interact with the cells/tissues [82]. 

### 3.1. Carbon Nanotubes

Carbon nanotubes (CNTs) can be thought of as long, slender fullerenes, where the walls of the tubes are hexagonal carbon (sp^2^ hybridized) and often capped at each end [105]. A typical categorization of CNTs takes into account the number of the walls constituting the nanostructure. Therefore, we can distinguish among single-walled (SWCNTs), double-walled (DWCNTs) and multi-walled carbon nanotubes (MWCNTs), whose properties are found to vary depending on their nature and chirality [106,107,108].

A SWCNT is formed by a graphene layer rolled-up along a given axis, defined as lattice vector, whose components determine the two key parameters of a nanotube, i.e., diameter and chirality [105]. In fact, depending on the chirality (i.e., the angle between hexagons and the tube axis), SWCNTs displaying the same diameter can be either metals or semiconductors, with band gaps that can vary up to 2 or 3 orders of magnitude [105]. For nanotubes displaying the same chirality, the band gap is inversely proportional to the diameter. Thus, each nanotube could display distinct properties [105].

Regardless of the intrinsic differences among various types of CNTs, they possess superior mechanical, thermal and electric properties. In fact, they show an elastic modulus close to that of pure diamond (around 1 TPa), thermal stability up to 2800 °C in vacuum, thermal conductivity about twice as high as diamond, electric-current-carrying capacity 3 orders of magnitude higher than copper wires [105]. Furthermore, electrical conductivity of CNTs may vary (up to 2 orders of magnitude) under mechanical bending or strain, and this electromechanical behavior is fully reversible [105]. 

Moreover, the recent use of CNTs for biological applications has made the development of several functionalization routes—including bio-functionalization—necessary, aiming at improving the interactions between biological molecules and nanomaterials. In fact, the cytotoxicity of CNTs is a key-issue still unchallenged, since their 1D geometry (similar to asbestos) and the strong hydrophobicity pose several issue in terms of biocompatibility. Surface modification of CNTs represent a successful strategy to tailor both bioactivity and dispersability of nanotubes but, conversely, some approaches may affect the electrical conductance of CNTs, due to the introduction of sp^3^ discontinuities within the sp^2^ framework [109,110]. Among the functionalization routes, covalent and non-covalent pathways were proposed, either in the presence or absence of solvents [110,111,112,113]. The choice of the type of derivatization obviously depends on the target application since, for instance, in nerve tissue engineering the electrical properties are crucial whereas for repairing other kinds of tissue one can prefer to covalently introduce hydrophilic moieties or even bioactive compounds to remarkably reduce the risks of cytotoxicity [92].

### 3.2. Graphene-Based Nanocarbons

**Figure 4:**
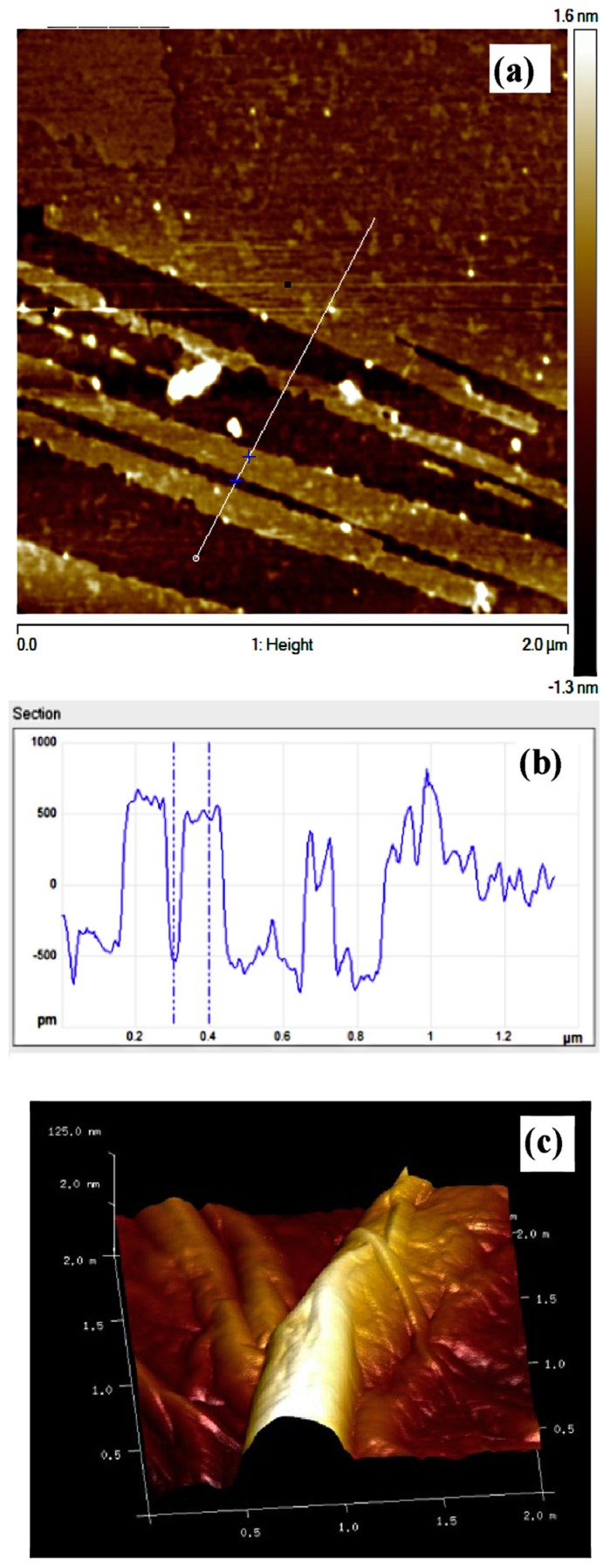
AFM images of the as prepared graphene oxide (GO) sample. (**a**) top view; (**b**) height profile of the region marked by the white line through the crosses in panel (**a**); (**c**) 3D view evidencing the wrinkling size. Reprinted from [118] with permission from Elsevier.

The definition of graphene refers to a one-atom thick honeycomb-like carbon sheet [79]. However, it is often present even under a few-layered form, being indicated as graphene nanoplatelets (GNP) or graphene nanosheets (GNS) [114]. Graphene, as well as GNP and GNS, are characterized by the strong prevalence of sp^2^ hybridized atoms, which result in a 2D planar, aromatic structure [114,115]. GNP, GNS and their multilayered counterpart, i.e., graphite, can be exfoliated into graphene by using organic solvents [116] or oxidized into graphene oxide (GO) [80]. This latter one, featuring a double honeycomb, constituted by the tunable presence of both sp^2^ and sp^3^ carbons, as well as aromatic and oxygenated domains, attracted enormous interest especially for biological applications [117]. In fact, the biocompatibility of graphene-based materials was found to increase upon increasing the hydrophilicity, that is the O/C ratio [92]. Highly oxygenated samples of GO were found to ensure good cytocompatibility and to promote cell adhesion, signaling and differentiation, presumably owing to the combination of hydrophilic moieties and wrinkled texture, since the presence of oxygen-containing functional groups are found to deform the graphenic planar lattice into a crumpled sheet-like configuration, as visible in Figure 4 [118,119]. 

Recent studies have been focused on demonstrating strong antimicrobial and antioxidant activities of GO, thus paving the pathway to the development of multifunctional biomaterials [92]. Of course, the presence of either aromatic and oxygenated groups make the GO lamellae highly dispersible in many solvents and provide the possibility to easily derivatize GO with a wide range of compounds in order to meet different demands [117]. Although the biocompatibility of graphene-based materials is found to increase as a function of O/C ratio, the mechanical and electrical properties are found to decrease upon increasing oxygen content. A defect-free graphene nanosheets possesses an elastic modulus equal to about 1 TPa, whereas it may decrease up to 200 GPa for GO [26]. Analogously, the electrical conductivity of defect-free single layer graphene is 10^4^ S/cm (at room temperature) whereas that of graphene oxide is approximately 10^−1^ S/cm. However, it has to be taken into account that the amount of defects, as well as the mean dimension of lateral size and the content and type of oxygen functionalities may vary, depending on the kind of graphite source and the oxidation method used, with obvious influences on final properties of GO flakes. It is worth noting that GO can be even treated with reducing agents and converted into reduced graphene oxide (RGO), which physical-chemical properties are intermediate between those of graphene and GO.

### 3.3. Other Nanocarbons for Tissue Engineering

Among the other forms of nanocarbon materials currently employed in the fabrication of electrospun mats for tissue engineering, few papers report on the use of fullerenes and nanodiamonds. 

Fullerenes are cage-like structures of carbon atoms comprising hexagonal and pentagonal faces [120]. The first type of fullerene discovered was the C60 molecule, i.e., a hollow sphere composed by 60 carbon atoms where each side of a pentagon coincides with the adjacent side of a hexagon, thus being the nanosized analogous of a soccer ball [120,121]. Fullerenes show an extremely strong reactivity, with characteristics close to those of alkenes [121]. Indeed, they are involved in a wide variety of reactions, such as cycloaddition, nucleophilic and electrophilic substitution, thus being relatively easy to be functionalized [121]. In this context, an emerging trend is currently focused on the design and development of water-soluble fullerenes, particularly promising for studying the cellular uptake within the scaffolds, as well as the biodistribution, and even to perform organ/target binding tests. Among the fullerenes, those containing fluorescent nanoparticles have offered a high potential for bioimaging application due to their unique properties in terms of fluorescence emission, excellent solubility in water, good cell permeability, and high biocompatibility [104]. 

Nanodiamonds (ND), i.e., nanoscale diamond particles, are gaining a significant concern for many biological applications and only in the latest years, they are being considered as promising fillers for the fabrication of tissue engineering scaffolds.

The structure of NDs mainly depends on the technique used for their preparation. When NDs are achieved by the destruction of bigger (natural or artificial) diamond crystals, they display the same surface features as their bulk counterparts, whereas those obtained via detonation possess significantly different features [122]. In fact, the drastic conditions of the detonation environment lead to a large variety of surface functional groups on the particle surface. Furthermore, during this process re-graphitization phenomena may occur and they are usually prevented by using a cooling gas (CO_2_, H_2_O or inert gases), which obviously interacts/reacts with dangling bonds of NDs, thus influencing surface chemistry of the resulting nanoparticles [122]. As a consequence, the sp^2^/sp^3^ ratio in NDs may extremely vary, as already seen for GO. The bare (non-functionalized) surfaces of cubic crystals exhibit structures similar to bulk diamond, whereas the surfaces of octahedral, cuboctahedral and spherical clusters exhibit a transition from sp^3^ carbon to sp^2^ carbon (re-graphitization) [103].

NDs display a variety of surface functionalities, particularly suitable to adsorb or graft functional groups or much more complex moieties, for example, proteins or DNA, onto their surface. Differently from graphene and carbon nanotubes, NDs dispersions show strong colloidal stability in aqueous or polar media [103]. Furthermore, good biocompatibility and low cytotoxicity enable their use in a broad range of biological applications. Indeed, when they are used as fillers for tissue engineering applications, they can endow the resulting scaffolds with additional properties or functions, such as adsorptive separation, purification and analysis of proteins, vehicles for drugs, genes and antibodies and fluorescence labeling [122]. Shin and coworkers demonstrated the possibility to stimulating the myogenesis of C2C12 myoblasts via the incorporation of GO into PLGA either decorated with a peptide (RGD-peptide) [123] and hybridized with collagen [124]. When GO is added to PLGA/RGD matrix, the cell adhesion and proliferation is ensured by RGD peptides, whereas GO serves as promoter for myoblast differentiation [123]. 

## 4. Electrospun Polymeric Nanomats Containing Nanocarbons for Tissue Engineering

### 4.1. Polymeric Nanomats Containing Carbon Nanotubes

The use of CNTs in combination with biocompatible polymers offers attractive properties that make them suitable for biomedical applications. Attempts in this sense rely on the use of CNTs as reinforcements or additives to improve material physicochemical properties (e.g., strength, stiffness, electrical conductivity) or to achieve new functionalities. Moreover, the use of polymers allows the achievement of a wide collection of mechanically stable scaffold structures while reducing CNT cytotoxicity in the resulting materials [125]. Indeed, electrospun scaffolds containing CNTs have been extensively explored for TE applications, particularly for the regeneration of neural [125,126,127,128,129], muscle [35,130,131,132] and bone tissues [133,134,135] as reported in Table 2. Several polymers have been used as matrix of electrospun mats, including natural polymers such as gelatin [131] and silk [135,136]. Furthermore, the applicability of synthetic polymers for CNT dispersion has been also exploited, including polycaprolactone (PCL) [35,137], polyurethanes (PU) [138,139,140], poly(lactic-*co*-glycolic acid) (PLGA) [126,127,132], and especially polylactic acid (PLA) [125,129,134,141,142,143]. In this context, the functionalization of pristine CNTs could represent a key step for the preparation of CNT-nanomats [125]. Indeed, the decoration of the pristine nanotubes with organic moieties allowed overcoming the main problem related to their use as nanofillers in polymer matrices, which is the pronounced tendency to form aggregates and bundles, because of the strong van der Waals mutual interactions between their sp^2^-carbon networks. In addition, from a toxicological perspective, functionalization can prevent the formation of intracellular aggregates, i.e., potentially dangerous structures, in case some cell eventually incorporate CNTs released from polymer matrix.

**Figure 5:**
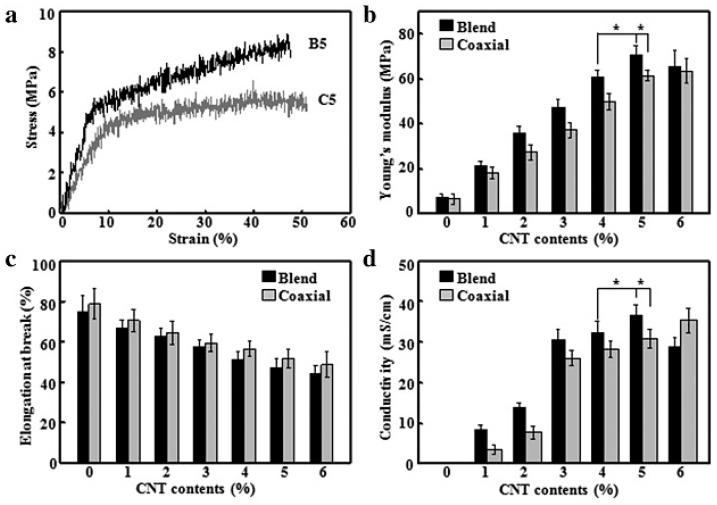
(**a**) Typical stress–strain curves of blend and coaxially electrospun fibrous mats containing 5% of carbon nanotubes (CNTs). (**b**) Young’s modulus, (**c**) elongation at break and (**d**) conductivity of blend and coaxially electrospun fibrous mats containing different amounts of CNTs. Reprinted from [130] with permission from Elsevier.

The fiber morphology and topography as well as the incorporated CNT amount can play a crucial role in tuning the mechanical and electrical properties and consequently the biocompatibility of polymeric nanomats containing CNTs [130,134]. Generally, electrospun aligned mats display higher mechanical properties in comparison with random ones [134,135,144]. Moreover, the alignment can promote the proliferation of specific cells [134]. Regarding the CNT amount, usually, on increasing the CNT loading, the mechanical and electrical properties can dramatically improve, as visible in Figure 5, which reports the main results related to blended and coaxially electrospun fibrous mats containing different amounts of CNTs [130]. In particular, Young's modulus (Figure 5b) significantly increased up to 5% of CNTs, while when the CNT content reached 6%, there was no apparent change for coaxial fibers and a slight decrease for blend fibers, due to the filler re-aggregation. The elongation at break of mats (Figure 5c) was found to decrease from around 75% to 45% upon increasing the CNT concentration both in blend and coaxial fibers, because of the stiffening effect of CNTs. The conductivity of fibrous mats (Figure 5d) as a function of CNT content displayed the same behaviour as Young’s modulus. In fact, electrical conductivity of coaxial fibers increased linearly with CNT content, whereas in the case of blended system this property reached a maximum when CNT content was equal to 5%, thereafter it was found to decrease, presumably due to re-aggregation phenomena. However, for both systems the electrical percolation threshold was observed at around 3%.

The diameter of electrospun fibers can depend on the CNT concentration [130,134]. In particular, thinner fibers are usually obtained with the increase in the CNT contents due to the increased conductivity of electrospinning suspensions [130].

#### 4.1.1. Natural Polymers

Silk as a matrix for CNT-mats was used by Pan et al. [136], who obtained microcomposite fibers from regenerated silk fibroin and functionalized MWCNTs by an electrospinning process from aqueous solutions. The mechanical properties of the reinforced mats were greatly improved by incorporating MWCNTs up to a loading level of 1%. Thereafter, the critical aggregation of MWCNTs negatively affected the ultimate properties of the resulting materials. Preliminary tests demonstrated that the electrospun fiber mats have good biocompatibility for tissue engineering scaffolds. In particular, the results indicated that mats had no obvious cytotoxicity for attachment, growth, and proliferation of 3T3 cells and lingua mucosa cells.

The use of SWCNTs for the fabrication of electrospun scaffolds for tissue engineering has been more rarely accomplished whereas, the use of MWCNTs has been more extensively pursued. For instance, SWCNTs were used to fabricate nanocomposite silk fibers by co-electrospinning [135]. In particular, Gandhi et al. [135] successfully electrospun regenerated silk protein from cocoons of *Bombyx mori* producing random as well as aligned nanofibers containing 1% wt of SWCNTs. Adding CNTs significantly increased some crucial properties of silk fibers, including tensile strength, toughness and especially electrical conductivity (+400%).

Ostrovidov et al. [131] fabricated aligned electrospun gelatin-MWCNTs nanofibrous scaffolds for the growth of myoblasts. The MWCNTs significantly improved myotube formation by enhancing mechanical performance and upregulated the activation of the genes related to the mechanic transduction. In particular, a significant increase in myotube length when MWCNTs were integrated in the nanofibers was observed. Furthermore, with increasing the MWCNTs content the myotube length increased, reaching, for the highest content, values 320% higher than that of myotubes formed on gelatin fibers without carbon nanotubes.

#### 4.1.2. Synthetic Polymers

PLA and related copolymers are frequently used as synthetic matrices for electrospun mats for tissue engineering, owing to good biocompatibility, adjustable degradation rate, ease of processing and excellent mechanical properties of these polymers, further enhanced by the incorporation of CNTs, even at low concentrations [125,126,127,129,132,134,141,142,143]. 

Shao et al. successfully fabricated random oriented and aligned PLA/MWCNTs nanofiber meshes by electrospinning [134]. They showed that average diameter of nanofibers can be controlled by adjusting the amount of MWCNTs. Moreover, the incorporation of CNTs strongly enhanced both the mechanical and electrical properties. Furthermore, these conductive nanofibrous scaffolds paved the way to study the synergistic effect of topographic signals and electrical stimulation on osteoblasts growth, with potential applications in bone tissue engineering. The results showed that the aligned nanofibers were more efficient than their random counterparts in osteoblasts signaling and directioning.

**Figure 6:**
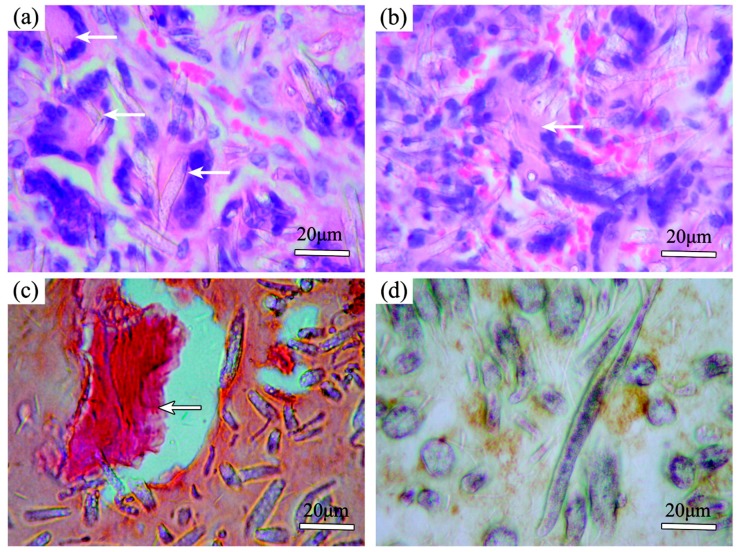
Histologic examination of cell/membrane composites implanted into immunodeficient mice: (**a**–**c**) show new-formed bonelike tissues in round or irregular shape (white arrow), and osteoblast-like cells were well arranged around bonelike tissues. Abundant blood vessels were found in the implanted area. In (**c**), alizarin red staining confirmed calcium deposits in new-formed bonelike tissues. In (**d**), osteocalcin, which was stained in brown, was detected in the cytoplasms and outside the cells. Reprinted with permission from [143]. Copyright (2007) American Chemical Society.

Mei et al. developed an electrospun random mat consisting of PLLA, MWCNTs and hydroxyapatite (HA) to satisfy the specific requirements of a guided tissue regeneration (GTR) membrane [143]. In particular, they found that the presence of the CNTs improved the selectivity of the membrane, thus promoting the adhesion and proliferation of periodontal ligament cells (PDLCs) while inhibiting the adhesion and proliferation of gingival epithelial cells. Therefore, PLLA/MWCNTs/HA membrane seeded with PDLCs were implanted into the leg muscle pouches of immunodeficient mice. All animals survived without any local or general complications until the scheduled experimental time. Histologic examinations showed that PDLCs attached on the membranes functioned well in vivo and no obvious inflammation was found in the implant areas. Representative microscopy photographs of paraffin sections that underwent histologic examinations and immunohistochemical staining for osteocalcin are reported in Figure 6 Bone-like tissues were formed with a round or irregular shape and were stained into homogeneous pink by hematoxylin/eosin, and osteoblast-like cells were well-arranged around the bone-like tissues. Calcium deposits were confirmed in new-formed bone-like tissue by alizarin red staining. Moreover, abundant blood vessels were grown into the new formed tissues. Osteocalcin, which was stained in brown, was detected in the cytoplasm and outside the cells.

Vicentini et al. reported a study on the use of 4-methoxyphenyl functionalized MWCNTs as nanofiller into a PLLA matrix for the preparation of electrospun fibrous scaffold boosting neurite outgrowth and neuronal cell differentiation [125]. The tailored covalent functionalization of nanotube surfaces allowed a homogeneous dispersion of the nanofillers within the polymer matrix, diminishing their natural tendency to aggregate and form bundles. Furthermore, TEM images showed carbon nanotubes anisotropically aligned along the fiber axes. The scaffolds prepared were tested in terms of biocompatibility and neuritogenesis and those containing CNTs gave the best results in neurite outgrowth, likely due to the nanocarbons-induced neuronal differentiation.

Other papers reported studies on the use of lactide polymers and CNTs for the fabrication of devices useful for neural tissue engineering [126,127,129]. In particular, Edwards et al. combined the properties of PLGA and MWCNTs not incorporating the nanofiller into the polymeric fibers, but electrospinning PLGA nanofibers onto a tubular MWCNT knitted scaffold [126]. 

In this case, the presence of electrospun PLGA led to the formation of small pores that enabled the spanning and uniform distribution of cells, thus avoiding the formation of cell clusters irregularly distributed on the surface, otherwise found in knitted tubular scaffolds only.

**Figure 7:**
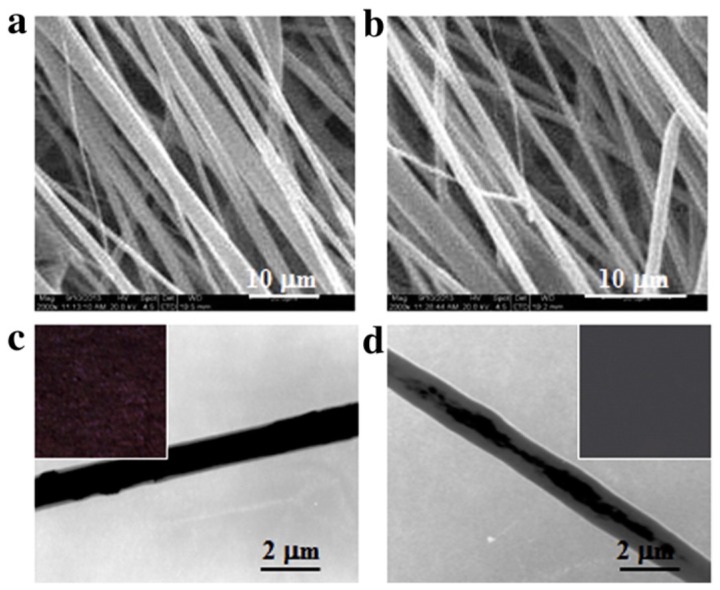
(**a**,**b**) Typical SEM and (**c**,**d**) TEM images of blend (**a**,**c**) and coaxially electrospun fibers (**b**,**d**) containing 5% CNTs. Insets in c and d show the physical appearance of fibrous mats obtained. Reprinted from [130] with permission from Elsevier.

An alternative way to incorporate an even higher amount of CNTs into electrospun mats but avoiding their cytotoxicity is entrapping CNTs in fiber cores through coaxial electrospinning [35,130]. Liu et al. prepared fibers of poly(ethylene glycol)-poly(d,l-lactide) copolymers (PELA) containing up to 6% of CNTs by blend and coaxial electrospinning to create a synthetic microenvironments to improve the function of cardiomyocytes [130]. The electrospun mats were collected on a rotating mandrel thus obtaining highly aligned fibers as shown by SEM micrographs reported in Figure 7a,b. TEM images of fibrous mats reported in Figure 7c,d show a bulk distribution of CNTs in blend fibers, whereas coaxially electrospun fibers exhibit a core-sheath structure with the embedment of CNTs in the fiber cores. Due to the preferred location of CNTs in the fiber cores packed by PELA sheath, coaxial fibers were gray (inset of Figure 7d), while blend electrospun fibers appear black (inset of Figure 7c). The biological results revealed that higher loading amount of CNTs in fibers maintained the cell viabilities, induced the cell elongation, enhanced the productions of contractile proteins, and promoted the synchronous beating behaviors of cardiomyocytes. Moreover, although the conductivity of blend fibers was slightly higher than that of coaxial fibers with the same CNT loadings, the lower exposures to CNTs due to their entrapment in fiber cores resulted in higher cell viability, elongation, extracellular matrix secretion and beating rates for cardiomyocytes on coaxial electrospun fibers.

The incorporation of MWCNTs into conducting polymers such as polyaniline [145,146] gave rise to electrospun fibers suitable as scaffolds in cell culture studies. Indeed, the presence of CNTs improved cell growth and proliferation on the surface of the conducting nanofibers because of their conductivity and mechanical strength provided by the PANI and CNTs.

Rodrigues et al. [133] proposed the use of electrospun poly (butylene adipate-*co*-terephthalate) (PBAT)-based fibers for bone regeneration, in spite of the poor mechanical resistance of neat PBAT. The authors demonstrated the possibility to overcome this drawback by adding low contents of superhydrophilic MWCNTs (0.1–0.5 wt %), owing to their remarkable strengthening and stiffening effect. All samples showed cytocompatibility with MG63 osteoblast-like cells and in particular, on increasing the MWCNTs content increased the cellular viability, thus indicating that the incorporation of 0.5% of MWCNTs increased its biocompatibility. Moreover, MG63 cells osteogenic differentiation showed that mineralized nodules formation was increased in PBAT/0.5% MWCNTs when compared to control group and neat PBAT.

Another possible way to combine the properties of electrospun mats and CNTs, different from the conventional incorporation, is the nanofiller coating on the surface of the nanomats, as proposed by Jin et al. [128]. In particular, PLCL electrospun fibers were coated with ad hoc functionalized MWCNTs in order to provide better environments for cell adhesion and neurite outgrowth. The results revealed that MWCNT-coated PLCL scaffolds exhibit improved adhesion, proliferation and neurite outgrowth of PC-12 cells in comparison with uncoated PLCL scaffolds.

### 4.2. Polymeric Nanomats Containing Graphene-Based Nanocarbons

The main works on electrospun nanomats containing graphenic compounds for tissue engineering are listed in Table 3. Among the graphenic compounds, GO is the most widespread one, because of its better biocompatibility. In fact, its unique chemical-physical features, such as the high hydrophilicity ensured by the presence of a wide range of oxygenated moieties and the wrinkled texture that results in a high roughness, are useful to provide cell proliferation and attachment, respectively. The choice of the matrix for GO-containing electrospun mats mainly depends on the target tissue for which the materials are proposed. Naturally derived as well as synthetic biodegradable polymers are the most widespread. The former enable high degrees of cells adhesion, the latter provide better mechanical performance. 

Lactide polymers, such as PLA, PLLA, PLGA, are frequently used for bone tissue engineering, owing to the excellent mechanical properties of these polymers, further enhanced by the incorporation of GO, even at small concentrations. Moreover, GO allows increasing surface wettability of PLA, which is found to change from a hydrophobic to hydrophilic character, with positive repercussions on cell adhesion and proliferation. 

As previously discussed, the mean diameter of the nanofibers can be tuned by varying several processing parameters. For the system PLA-GO, the nanofibers diameter distribution was found to vary from hundreds of nanometers to few microns. The PLA-GO bionanomats for bone tissue engineering are composed by randomly oriented nanofibers, since these particular structures provide attractive ECM conditions for the anchorage, migration and differentiation of tissue cells, including those responsible for the regeneration of bone [149]. Moreover, GO was found to promote both cell signaling and differentiation due to its wrinkled texture [150]. 

#### 4.2.1. Natural Polymers

Massoumi et al. prepared electrospun nanofibrous scaffolds based on gelatin and a functionalized GO [151]. The authors covalently attached a copolymer (poly(2-hydroxyethyl methacrylate)-graft-poly(ε-caprolactone)) onto an acylated sample of GO via atom transfer radical polymerization (ATRP). The electrical conductivity of the electrospun nanofibers obtained was in the scale of 10^−5^ S/m, which represents proper conductivity for scaffolds addressed to repair injured nerve tissues [151]. 

**Figure 8:**
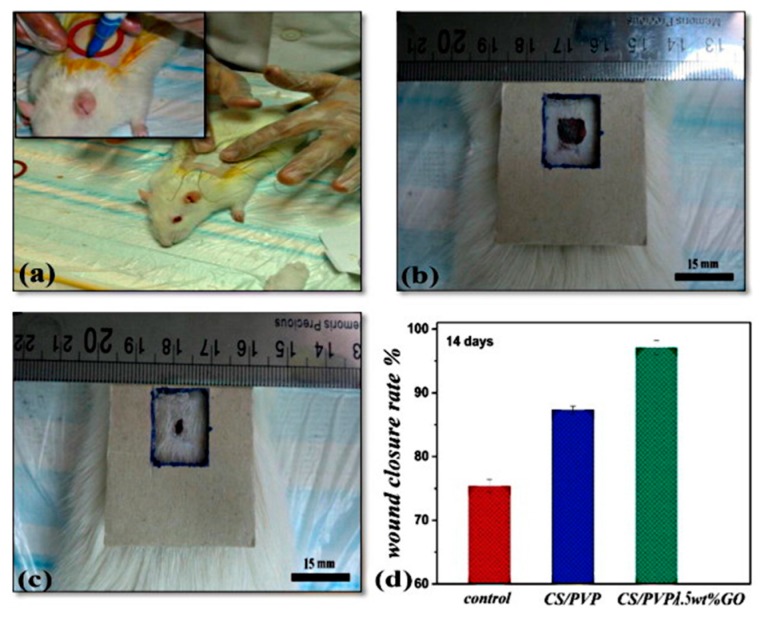
Electronic images show (**a**) surgery process of implantation of nanofibrous membranes on the open wound of a rat, and wound healing 14 days post-surgery for (**b**) pristine chitosan (CS)-based mat and (**c**) 1.5% GO-containing membrane. (**d**) Wound closure rate for the examined materials compared with the control (sterile gauze sponge). Reprinted from [152] with permission of Elsevier.

Nafiseh and Simchi fabricated nanofibrous scaffolds by electrospinning blended solutions of chitosan (80 vol %), polyvinyl pyrrolidone (15 vol %), polyethylene oxide (5 vol %) containing GO nanosheets (0–2 wt %) [152]. GO significantly increased the conductivity and viscosity of highly concentrated chitosan solutions, thus enabling the spinnability of ultrafine and uniform fibers with an average diameter of 60 nm. The GO-reinforced nanofibers displayed enhanced elastic modulus and tensile strength (150%–300%) with a controllable water permeability to meet the required properties of natural skins. Furthermore, the nanofibrous structure was found to promote the cell attachment, by maintaining characteristic cell morphology and viability up to 72 h. The nanofibrous membranes based on neat CS and containing 1.5% GO were implanted on open wounds, as shown in Figure 8a. In vivo evaluations in rats showed a faster and more efficient wound closure rate in the case of nanofibrous membranes and those loaded with 1.5% GO gave the best results, as clearly visible by comparing the electronic images of the examined rat and the area of open wound after 14 days post-surgery in the case of neat CS (Figure 8b) and CS containing 1.5% GO (Figure 8c). The wound closure rate was evaluated by image analysis and shown in Figure 8d. The polymeric nanofibrous membrane promoted the healing process compared with the control (sterile gauze sponge). This feature was attributed to the ultrastructure of the dressing materials together with the inherent healing abilities of CS for open wounds. GO doping results in a further enhancement of wound closure ability (about 33% as compared with sterile gauze sponges). The presence of GO nanosheets led to further advantages, including higher strength, adapted permeability, better cell attachment, and total absence of scar and/or inflammation owing to its antibacterial activity.

Azarniya et al. integrated GO within chitosan (CS) and bacterial cellulose (BC), aiming at fabricating electrospun nanofibrous scaffolds for skin tissue engineering [153]. An enhancement in tensile strength and elastic modulus (40% and 115% increase, respectively) with about 60% decrease in elongation was measured for the 1.5% GO-reinforced nanocomposite as compared with the pristine CS/BC nanofibers. The addition of the GO nanosheets was accompanied with a gradual decrease of hydrophilicity. Furthermore, the presence of GO (1.5%) halved the water vapor permeability of the CS/BC nanofibers.

Aznar-Cervantes et al. coated an electrospun silk fibroin nanofibrous mat with GO to prepare multifunctional nanohybrids for biomedical applications, ranging from osteochondral to nerve tissue repair [154]. GO-coating of the nanofibrous mat was achieved by electrochemical deposition with subsequent in situ reduction of GO into RGO by using ascorbic acid. The tensile tests performed onto matrix and nanocomposites containing either GO or RGO put into evidence that the coating with graphenic compounds reduced the capacity of elongation of the SF electrospun fibers, whereas the elastic moduli and ultimate strength were found to increase at low contents of GO and at high contents of RGO, respectively. Among the samples prepared, those containing GO displayed the best results in terms of fibroblasts proliferation within 9 days, whereas the RGO-coated meshes exhibited the highest electrical conductivity, thus being more suitable for nerve tissue engineering purposes. In this context, the electroactivity of the SF/RGO electrospun materials allows the performance of in vitro studies under electric fields or under ionic pulses. Indeed, the cell cultures can be subjected to constant or pulsating local electric fields inside the potential window of the electrolyte discharge, without current flow. 

Gao et al. prepared electrospun gelatin/CS/HA nanofibrous scaffolds reinforced with either GO or RGO to investigate the feasibility of fabricating materials gathering antibacterial properties and protein adsorption capability [155]. The antibacterial activity against Escherichia coli and Staphylococcus albus was greatly enhanced by GO, followed by RGO. Moreover, the GO-containing fibers displayed a good adsorption capacity of BSA at the normal physiological environment of the human body, thus being considered as promising materials for broad implications in the field of tissue engineering. PVA and GO were electrospun with small percentages of CS in order to gather the excellent biocompatibility and antimicrobial activity of GO and CS with the easy spinnability of PVA, which allows using water as a solvent. In this context, Liu et al. added GO to a PVA/CS nanofibrous mat, by achieving remarkable improvements in terms of mechanical properties and antibacterial activity, thus enabling PVA/CS/GO nanofibers as a promising candidate material in tissue engineering, wound healing and drug delivery system [156].

#### 4.2.2. Synthetic Polymers

Liu et al. prepared electrospun mats based on PLA, HA (which content was kept constant at 15 wt %) and GO, showing that the addition of small amounts of GO (from 1 to 3 wt %) caused a dramatic stiffening and strengthening of PLA/HA [162]. The authors reported that the mechanical properties were found to increase upon GO content up to a loading level of 2%. Indeed, the nanomats containing 2 wt % GO displayed values of elastic modulus and tensile strength practically two-fold with respect to PLA or PLA/HA, whereas those containing 3% GO showed mechanical properties worse than neat polymer. This issue is mainly due to the extremely high aspect ratio of GO, which tends to self-aggregate and crumple after a certain percolation threshold, as already evidenced by several studies performed onto different matrices [119]. From a biological point of view, the combination of HA with GO, especially when GO is loaded at 1%, exerts a remarkable effect on the adhesion and long-term proliferation of osteoblastic cells on the fibrous scaffolds. Furthermore, the authors demonstrated that GO acts synergistically with calcium phosphate, thereby increasing alkaline phosphatase activity and the calcium deposition of osteoblasts [162].

PLGA has been extensively investigated in the biomedical field because of excellent biocompatibility, biodegradability and processability, since glycolic acid units endow higher hydrophilicity and ductility than PLA. However, its mechanical stiffness is unsatisfactory for bone tissue engineering. Incorporating GO could enable the possibility of achieving structurally stable nanocomposites, capable to support osteoblast growth and proliferation. Luo et al. prepared electrospun nanofibrous scaffolds based on GO-doped PLGA. The results highlighted that—even if bioactivity of nanocomposites was found to be higher than neat copolymer—the mechanical performance was found to decrease [164]. The authors ascribed this issue to the 2D topological plane structure of GO, which is supposed to tend to be vertical to the fibers, thus being unable to transfer the stress [164]. However, it has to be taken into account that polyesters undergo a such rapid hydrolytic degradation in acidic environment and in some cases GO might have a pro-degradative effect, as reported for similar systems [7,171]. Indeed, depending on the synthesis conditions and the type of graphite source, some properties of GO, including acidity, C/O ratio (i.e., overall oxidation level) and relative surface content of –COOH and epoxy moietiesis, may vary considerably [7,171].

In some cases, PLGA was used in combination with natural polymers for GO-containing electrospun fibrous scaffold addressed to tissue engineering [123,124,165]. 

**Figure 9:**
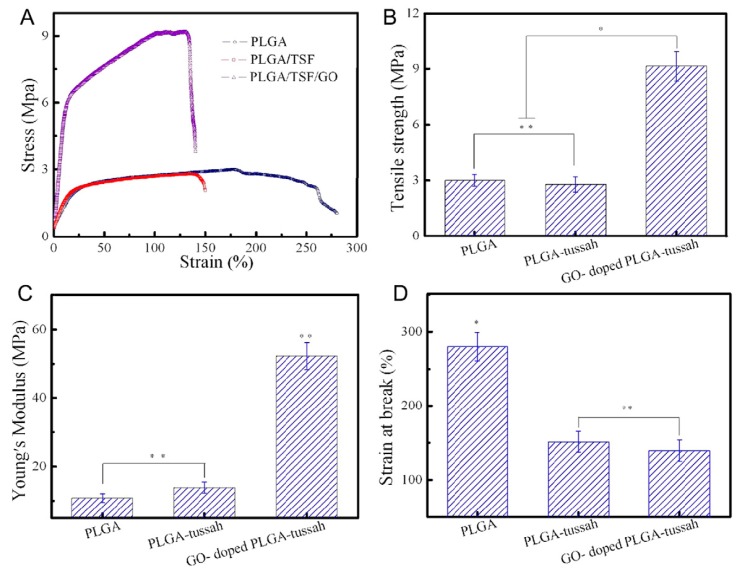
Mechanical properties of the electrospun fibrous pure poly(lactic-*co*-glycolic acid) (PLGA), PLGA–tussah, and GO-doped PLGA–tussah mats (*n* = 10 for each type of nanofibers) tested at room temperature. (**A**) Typical stress–strain curves; (**B**) tensile strength; (**C**) Young’s modulus; and (**D**) strain at break (* *p* < 0.05, ** *p* < 0.01). Reprinted from [165] with permission from Elsevier.

Shao et al. incorporated GO into a blend made of PLGA and SF in order to develop electrospun nanomats for bone tissue engineering. The results put into evidence that 1 wt % GO led to the simultaneous enhancement of mechanical and biological properties of the material. Figure 9 reports the representative tensile stress-strain curves (panel A) together with tensile strength (panel B), elastic modulus (panel C) and elongation at break (D) of electrospun fibrous PLGA, PLGA/SF and PLGA/SF/GO. As one can see, if compared to PLGA or PLA/SF, elastic modulus and tensile strength of the materials containing GO were found to be 5-fold and 3-fold, respectively [165]. The authors attributed the increase of tensile strength to the strong interfacial interactions between GO and PLGA (mainly due to hydrogen bonding), whereas the enhancement observed in the modulus was ascribed to the highly specific surface area of nanosheets.

The addition of GO to a PLGA/collagen matrix dramatically increased the mechanical performance (up to +500%) and wettability, whereas mean diameter and roughness of the nanofibers were found to decrease [124]. Studies on the bioactivity were performed onto PLGA, PLGA/collagen and PLGA/collagen/GO nanomats and the presence of GO gave the best results either on cells initial attachment and cell proliferation within 7 days. 

A useful strategy to avoid pro-degradative effects of GO on PLA and to improve interfacial adhesion is the covalent functionalization of GO. Zhang et al. carried out a comparative analysis between GO and GO-*g*-PEG as fillers for electrospun PLA [28]. The authors reported that GO-*g*-PEG was more effective than GO in enhancing thermal stability, mechanical properties, wettability. Furthermore, due to the presence of PEG chains, GO-*g*-PEG was able to remain dispersed within the polymer matrix even at high loadings (i.e., 5 wt %), differently from the pristine GO, which well-known tendency to self-aggregate was already discussed. Furthermore, the PLA cytocompatibility, investigated by studies on cell proliferation within 10 days, remained substantially unaltered after addition of either GO or GO-*g*-PEG.

An et al. fabricated PLA/PU blends filled with 3 and 5 wt % GO [163]. The authors reported that the materials displayed excellent biocompatibility together with a strong antimicrobial activity, imparted by GO, thus being suitable for cartilage and bone repairing while simultaneously reducing the threat of chronic infection of surrounding tissues.

GO, especially at high loadings, endows the nanomats with electrical properties, which are very suitable for nerve/neuronal tissue engineering. When applied to neuronal injuries repair, GO is preferentially coated onto electrospun polymers by a post-electrospinning treatment [166]. This two-step technique creates a conductive surface, particularly attractive for neuronal cells while maintaining the bulky properties of the polymer and using extremely lower contents of conductive fillers with respect to bulk inclusion-based techniques. Zhang et al. produced aligned PLLA nanofibrous scaffolds that were then surface modified via aminolysis in order to provide the surface with hydrophilic moieties, such as −OH, −COOH and −NH_2_, able to ensure a stable GO-coating [166]. The GO-coated nanofibrous scaffolds enabled the proliferation of Schwann cells cultured on the structure. In this case, the alignment of PLLA fibers promoted the neurite growth along the nanofibers, thus resulting in high cytoskeleton directioning and differentiation. The presence of GO led to remarkable increments in terms of cell proliferation (+40%), neurite length (+60%) and neurite bearing cells (+50%).

**Figure 10:**
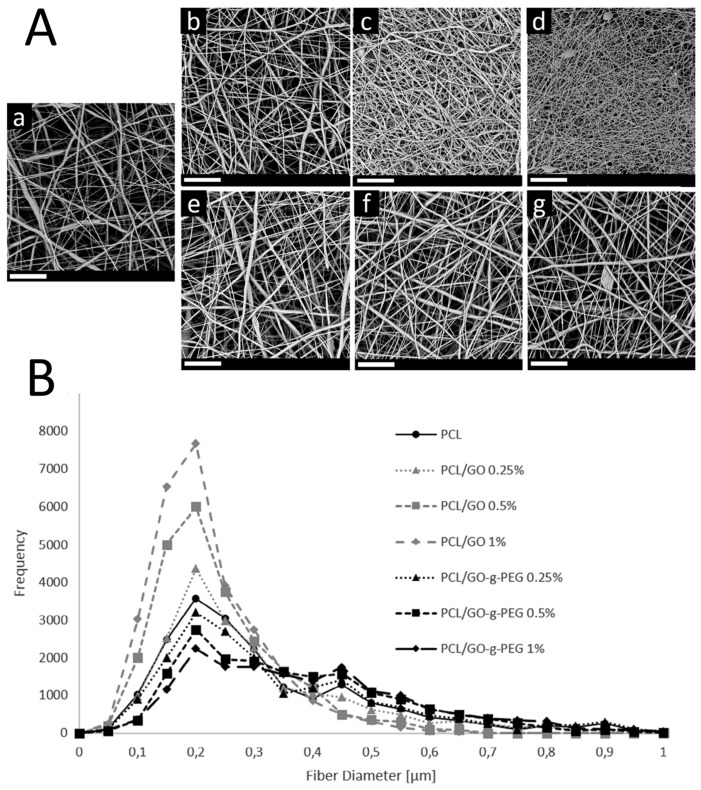
(**A**) SEM image of electrospun: (**a**) polycaprolactone (PCL); (**b**) PCL/GO 0.25 wt %; (**c**) PCL/GO 0.5 wt %; (**d**) PCL/GO 1.0 wt %; (**e**) PCL/GO-*g*-PEG 0.25 wt %; (**f**) PCL/GO-*g*-PEG 0.5 wt %; (**g**) PCL/GO-*g*-PEG 1.0 wt %. Scale bars are 20 μm. (**B**) Diameter distribution for electrospun PCL, PCL/GO and PCL/GO-*g*-PEG nanocomposites obtained with ImageJ. Reprinted from [7] with permission from Elsevier.

Another polyester commonly adopted for tissue engineering is polycaprolactone (PCL). Owing to its high resilience and ductility [172], PCL can be used either for cartilage and neuronal regeneration [8]. In this case, the addition of GO provides high strengthening and toughening, thus making PCL/GO nanocomposites suitable even for bone regeneration [160]. Indeed, systematic study on the effect of GO and RGO on the rheological and mechanical properties of electrospun PCL-based nanocomposites put into evidence that RGO provides better adhesion and nucleating effect than GO, presumably due to its higher hydrophobic character and its higher availability of sp^2^ domains that can interact with –CH_2_ moieties of PCL chains [161]. However, GO has higher bioactivity than RGO and for biomedical purposes GO is more suitable. Often, the GO is surface functionalized with other compounds or molecules in order to enhance its affinity to PCL without compromising its bioactivity [7,151]. Generally, the increments observed after addition of GO to a PCL matrix are dramatically higher than those reported for PLA-based nanomats. This feature mainly depends on the higher matrix-filler stiffness contrast [29]. Scaffaro et al. noticed that varying the loading level and the surface chemistry of GO enables the tuning of the mechanical properties of electrospun PCL-GO nanomats, thus allowing the prepared materials to be used either for bone and cartilage injuries by appropriately changing the preparation conditions [7]. The authors synthesized GO grafted with amino-terminated PEG moieties (GO-*g*-PEG), with the latter endowing more hydrophilicity and less acidity [81]. The increase of complex viscosity in PCL/GO-*g*-PEG suspensions caused an increase in the average fiber diameter if compared with PCL mats. In contrast, the complex viscosity of the PCL/GO suspensions was lower if compared with that of the PCL solution thus causing a reduction of PCL/GO nanofiber diameters, as visible from Figure 10A,B [7]. The authors related the reduction of the complex viscosity to the pro-degradative effect of GO due to the oxygenated functional groups of GO that can interact with the PCL chains [7].

GO-*g*-PEG was more effective in reinforcing PCL than GO, in particular at low concentration, likely due to the grafted PEG chains, which improved the filler dispersion. At higher filler loadings, instead, the presence of PEG determined a plasticizing effect, thereby enhancing the ductility. Moreover, biological tests demonstrated that PCL/GO-*g*-PEG enhanced the capability of supporting osteoblastic cells attachment and growth.

Mohammadi et al. demonstrated the effectiveness of PCL-GO nanomats to develop implants for bone tissue engineering [160]. In particular, the nanocomposite scaffolds containing 2 wt % of GO exhibited superior mechanical properties and faster biodegradability than pure matrix. Furthermore, the presence of GO promoted in vitro biomineralization, indicating bioactive features of the PCL-GO scaffolds, which showed better ability even in protein adsorption and in myoblasts adhesion and proliferation.

Atel et al. designed PCL-GO electrospun cell-instructive materials capable of controlling and guiding cellular behavior to promote skeletal muscle tissue regeneration [159]. The authors reported that the presence of GO, ranging from 0.3 to 2 wt %, increased wettability, hydration and electrical conductivity of PCL. However, the mechanical properties were found to be higher than those of PCL in nanocomposites containing 0.3% GO, whereas for higher loadings a slight worsening of elastic modulus and ultimate tensile stress was observed. Nevertheless, GO-containing nanocomposite scaffolds were found to promote myoblast differentiation without differentiation media and this property was found to monotonically increase upon GO content, as found by Chaudhuri et al. on an analogous system but with different fibroblasts [158].

Huang et al. integrated graphene nanosheets (GNS) into PVA nanofibrous scaffolds for cartilage tissue engineering [168]. The authors reported that the addition of the nanofillers increased the conductivity and viscosity of the PVA solution with opposite repercussions on fibers mean diameter that was found to decrease or increase, depending on which phenomenon prevailed on the other one. The PVA-GNS fibers were then thermally-treated to avoid rapid disintegration in water. The fibroblast cells proliferation was slightly increased by the presence of GNS. 

In contrast, by adding GO instead of GNS, the nanofiller incorporation plays an active role in cells adhesion and proliferation, together with a strong improvement in both stiffness and tensile strength up to a filler content of 1 wt %, as found by Qi et al. [169]. 

Jing et al. fabricated polyurethane (PU)/GO scaffolds via electrospinning at different GO contents as potential candidates for small diameter vascular grafts [167]. Tensile strength, Young’s modulus, and hydrophilicity of the scaffolds increased upon GO content. Biological essays were performed by using two types of cells, i.e., mice fibroblasts and human vein endothelial cells. Cell adhesion, proliferation and viability for both types of cells were the highest at a 0.5 wt % GO. Furthermore, an extensive characterization of these tubular scaffolds for their use as human blood vessels was carried out, by highlighting that tubular PU/GO scaffolds containing 0.5 wt % GO meet the requirements to be used in vascular tissue engineering. 

Interestingly, electrospinning was even used to generate a polymer template for achieving free-standing GO films for neuronal tissue engineering [170]. In their work, Jin et al. developed a novel fabrication method of free-standing reduced graphene oxide (RGO) films by vacuum filtering GO aqueous solutions through a nanofibrous membrane [170]. The GO was subsequently reduced into RGO and finally functionalized with fibronectin (FN). The presence of electrospun PVC mat endowed graphene films with a controllable patterning that increased the RGO roughness, particularly suitable to increase cell attachment and proliferation, due to the synergistic effects of the reactive functional groups in the FN protein and the large surface roughness and area of the graphene films. 

The same authors reported the fabrication of PAN/RGO nanofibrous scaffold by combining electrospinning process and chemical reduction [157]. PAN/RGO displayed high mechanical properties (tensile strain = 18.5% and tensile stress = 1.38 MPa). Biological test results showed that nanocomposites exhibited excellent biocompatibility, however very close to that of PAN, and high electrical conductivity, thus showing potential in the field of cell culture scaffolds for electrical stimulation.

### 4.3. Polymeric Nanomats Containing Other Nanocarbons

In the scientific literature, few papers report on the use of 0D nanocarbons for the fabrication of electrospun polymeric scaffolds for tissue engineering. Among these, nanodiamonds were added to PLA and PLGA for bone tissue repairs, and to chitosan for skin tissue engineering/wound healing [173,174,175,176], whereas water-soluble fullerenes were used to endow PLLA nanomats with fluorescent properties for bioimaging [104,177]. 

Nanodiamonds were integrated into a polymer solution and electrospun to develop a nanocomposite scaffold containing PLGA loaded with diamond nanoparticles [173]. The cytocompatibility experiments, carried out by seeding primary human mesenchymal stem cells (hMSCs) on the scaffolds, showed that addition of diamond nanoparticles did not affect cell proliferation, and no cytotoxic cellular response was detected within 9 days in culture. Nanoindentation measurements revealed the presence of higher hardness zones at certain points on the fibers of PLGA-nanodiamonds composites, likely owing to nanoparticles reinforcing mechanism. This study demonstrates that PLGA nanofibers can be reinforced with nanodiamonds without adversely affecting cell behavior, thus displaying a good potential for future application in bone tissue engineering. Parizek and coworkers [174] investigated the structure-property relationship of PLGA/nanodiamonds system in the case of high nanofiller loadings (23 wt %). The authors reported that ND particles were either found to be arranged in a bead-like configuration in the central part of the fibers or to form clusters protruding from the fibers. The presence of ND resulted in meshes constituted by thicker fibers (with a +25% relative increase in diameter), however displaying smaller areas of pores (−64%). The PLGA-ND samples showed higher mechanical performance and improved bioactivity on human osteoblast-like MG-63 cells, which were found to be polygonal or spindle-like in shape, and distributed homogeneously onto the membranes. Within 7 days after seeding, cell number was found to rise continuously, and at the end of the experiment, these cells were able to create a confluent layer. The cell viability ranged from 92% to 97% and no significant inflammatory activity was detected. 

Pereira et al. prepared electrospun nanomats based on PLA and low loadings of nanodiamonds (from 0.1 to 1 wt %) for bone tissue engineering [176]. Substantially, the results achieved are in good agreement with those reported for PLGA/ND systems: incorporating NDs into the biopolymer determined the enhancement of hydrophilicity with positive effects on cell adhesion and proliferation, while no cytotoxic effects were recognized in osteoblasts (L929 cells).

Mahdavi et al. [175] reported the successful electrospinning of chitosan-based biopolymers containing bacterial cellulose (33 wt %) and medical grade nanodiamonds (3 nm; up to 3 wt %) for skin tissue engineering. Morphological analysis put into evidence that introducing diamond nanoparticles facilitated the electrospinning process with a decrease in the size of fibers (from 180 to 70 nm) and a highly uniform distribution of diameters and lengths. The authors found that beyond 1 wt %, percolation networks of nanoparticles were formed thus improving the properties of the nanofibrous mats: the enhancement of tensile strength (from 13 to 25 MPa) and hydrophilicity (favorable for cell attachment), as well as the possibility to tune the water vapor permeability (from 342 to 423 μg·Pa^−1^·s^−1^·m^−1^) suggest that nanodiamonds-modified mats are potentially suitable for wound healing applications.

Liu et al. reported the preparation of PLLA nanofibers encapsulated with water-soluble fullerene nanoparticles by blend electrospinning [104]. The nanofibers were uniform, with average diameters ranging from 300 to 600 nm. Water-soluble fullerene nanoparticles were encapsulated within the nanofibers, forming a core–shell structure and thereby endowing the resulting nanocomposite mats with excellent hydrophilicity. The mechanical properties, although lower than those of the pure matrix, were however desirable to tissue-engineered materials. The in vitro biological imaging experiments indicated that fullerene nanoparticles were released from the nanofibers and penetrated into human liver carcinoma HepG-2 cells. The integration of water-soluble fullerene nanoparticles within PLLA nanofibers allows us to achieve fluorescent biomaterials that are particularly promising for applications in bioimaging and drug delivery.

The same authors prepared a similar system by adding paclitaxel [177]. The delivery tests performed in vitro demonstrated that the fullerene content greatly influenced the release rate of paclitaxel. Furthermore, while the drug paclitaxel inhibited the proliferation of HepG-2 cells effectively, the fluorescent signal of fullerenes in HepG-2 cells reflected the growth state of cells clearly. Hence, these ternary scaffolds could have a significant potential for tissue engineering, drug delivery and bioimaging applications.

## 5. Conclusions

Most of the electrospun scaffolds containing nanocarbons summarized in this review have proved to be good substrates for the adhesion, growth and differentiation of cells and stem cells. 

Among the nanocarbons, 1D CNTs and 2D graphene-based nanomaterials are the most widespread. The use of 0D nanocarbons, such as nanodiamonds and fullerenes, is less investigated. 

Generally speaking, either CNTs or graphene were used with a wide range of polymers, thus generally leading to a remarkable enhancement of mechanical and electrical properties. 

In particular, CNTs are successfully used, especially for repairing injured nerve tissues, since they possess a unique combination of high electrical conductivity and strong ability to align along the fibers. These two features are particularly required for neuronal and muscle tissue engineering applications. However, due to their cytotoxicity, often CNTs have to undergo derivatization reactions with hydrophilic moieties or biomolecules. 

Among the graphene-family, GO is very promising in regenerating cartilage, bone, skin and nerve tissues. In particular, due to its good cytocompatibility, GO can be used even without any further functionalization route and its surface texture offers a suitable environment for cells adhesion and proliferation, whereas its antimicrobial properties prevent the risk of inflammatory processes and its electrical properties are found to be suitable for neuronal cells. Conversely, due to their geometry, 2D lamellae display a strong tendency to re-aggregate after a certain concentration threshold, ranging from 2% to 5% weight content, depending on the processing conditions adopted, as well as host polymer properties.

On the other hand, nanodiamonds and fullerenes determine mechanical properties lower than CNTs and graphene but still desirable for certain applications, such as skin tissue repair. Nanodiamonds are mainly used to enhance the hydrophilicity of lactides, aiming at improving bioactivity of the scaffolds. 

Modified fullerenes are able to endow the scaffolds with additional properties, such as fluorescence, particularly useful for bioimaging, and controlled drug release. In fact, fullerenes display a broad-spectrum reactivity that allows achieving water-soluble, fluorescent nanocarbons. Moreover, they are promising as carriers for a wide range of bioactive agents, owing to their strong ability to penetrate into cells. They can be useful in the development of advanced scaffolds, where regionally selective cell adhesion and directed growth is required, and in genomics and proteomics.

## Figures and Tables

**Table 1:** Nanocarbons examined in this review: some properties of interest.

Nanocarbons for TE		Mechanical properties		Electrical properties			Biological properties	
Class and Geometry	Type	*E* (TPa)	TS (GPa)	CCM (cm^2^/V·s)	Band gap (eV)	Conductivity (S/cm)	Cytotoxicity	Antibacterial activity
CNT-family (1D)	SWCNTs	1–1.3 [83]	13–52 [83]	1 × 10^5^ [84]	0.01–0.5 [84]	10^2^–10^3^ [79]	Strong [85]	Strong [86]
	DWCNTs	1.25 [83]	45 [83]	1 × 10^5^ [84]	0.01–0.5 [84]	10^2^–10^3^ [79]	Strong [85]	Strong [87]
	MWCNTs	0.2–0.9 [88,89]	1.7 [83]	1 × 10^5^ [84]	0.01–0.5 [84]	10^2^–10^3^ [79]	Moderate [85]	Moderate [87]
Graphene family (2D)	Graphene	~1 [90]	130 [91]	2 × 10^5^ [91]	0 [91]	10^4^ [82]	High [92]	Moderate [93]
	GO	0.25–0.4 [90,91,94]	30–60 [90,91]	Var [95,96]	Var [95,96]	10^−1^ [82]	Low [92]	Strong [97]
	RGO	0.1–0.4 [94]	30–99 [90,91]	1 × 10^5^ [95]	0.01–0.05 [95]	10^2^–10^4^ [82]	Moderate [92]	Moderate [93]
Other nanocarbons (0D)	Fullerenes	N/A	N/A	6 [98]	1.5–2.3 [99,100]	10^2^–10^4^ [100]	Moderate [85]	N/A
	NDs	1–1.3	N/A	10^3^–10^4^ [101]	5.5 [102,103]	10^−2^ [102]	Low [104]	N/A

N/A: Not available; Var: Variable; CCM: Charge carrier mobility.

**Table 2:** Examples of polymer-CNTs electrospun scaffolds for tissue engineering.

Polymers (and additives)	Solvents	Nanocarbons	Nanocarbons loading (wt %)	Experimental setup	Structure	Main improvements	Target tissue	Refs.
CA/CS	Acetone/DMF (2:1)	MWCNT	N/A	electrospinning plus layer-by-layer self-assembly	Random, *D* = 305 ± 128 nm	Mechanical properties; cell attachment, spreading and proliferation	Not specified	[147]
Gelatin	Water	MWCNT	N/A	Electrospinning followed by crosslinking with GA vapor	Aligned, *D* = 296 nm	Mechanical properties; cell alignment and differentiation	Muscle	[131]
PANI/PNIPAm-*co*-MAA	HFIP/DMF (8:2)	PANI-MWCNT	N/A	Conventional electrospinning	Random , *D* = 500–600 nm	Cell growth and viability	Not specified	[145]
PANI/PNIPAm	HFIP/DMF (8:2)	HOOC-MWCNT	N/A	Conventional electrospinning	Random , *D* = 400–500 nm	Cell proliferation and viability	Not specified	[146]
PBAT	Chloroform/DMF (3:2)	MWCNT (plasma treated with O_2_)	0.1%–0.5%	Conventional electrospinning	Random, *D* = 250 ± 52 nm–272 ± 79 nm	Mechanical properties	Bone	[133]
PCL	DCM/methanol (3:1)	MWCNT (acid-treated)	0.1%–5%	Conventional electrospinning	Random, D = 117±45–252 ± 146 nm	Accelerating degradation behavior; biocompatibility	Not specified	[137]
PCL–PAA/PVA	DMF/DCM (1:1)–EtOH/H_2_O	MWCNT (acid-treated)	0.05%	Coaxial electrospinning	Random, *D* = 1.861 ± 0.693 μm	Mechanical and electrical properties; biocompatibility	Skeletal muscle	[35]
PELA	DMF/DCM	MWCNT	0%–6%	Coaxial electrospinning	Aligned, *D* = 2–3 μm	Mechanical and electrical properties; cell morphology	Myocardial	[130]
PLA	Chloroform/DMF	MWCNT	0%–1%	Conventional electrospinning	Random, *D* = 0.55–0.96 μm	Mechanical and electrical properties	Not specified	[141]
PLA	DCM/DMF (3:1)	MWCNT	1%	Conventional electrospinning	Random, *D* = 2.08 ± 0.13 μm	Mechanical and electrical properties	Cartilage	[142]
PLA	DMF/DCM	MWCNT (acid-treated)	0%–5%	Conventional electrospinning	Random, *D* = 243–425 nm Aligned, *D* = 232–402 nm	Mechanical and electrical properties; cell morphology	Bone	[134]
PLCL	DCM/EtOH (4:1)	MWCNT-tartrate	N/A	MWCNT coating on electrospun PLCL	Aligned, *D* = 1.30 ± 0.46 μm,	Cell adhesion, proliferation and neurite outgrowth	Nerve	[128]
PLGA	DMF/THF (3:1)	MWCNT	0.1%–1%	Conventional electrospinning	Random, *D* = 0.4–1.6 μm	Electrical properties; myotube formation	Skeletal muscle	[132]
PLGA	DMFA	MWCNT	N/A	electrospinning onto MWCNT knitted scaffold	Random *D* = N/A	Cell spanning	Nerve	[126]
PLGA/SF/catalpol	HFIP	MWCNT	N/A	Conventional electrospinning	Random, *D* = 577 ± 360–810 ± 270 nm	N/A	Nerve	[127]
PLLA	Chloroform/DMF (9:1)	MWCNT-PhOMe	0.25%	Conventional electrospinning	Random, *D* = 200–600 nm	Neurite outgrowth and neuronal cell differentiation	Nerve	[125]
PLLA	Chloroform/DMF (8.5:1.5)	SWCNT	3%	Conventional electrospinning	Aligned, *D* = 430 nm	Cell adhesion, growth, survival and proliferation	Nerve	[129]
PLLA/HA	DCM/1,4-dioxane	MWCNT (anodic oxidated)	0.3%	Conventional electrospinning	Random, *D* = 1 μm	Cell adhesion and proliferation.	Periodontal ligament	[143]
PU	THF/DMF (1:1)	MWCNT	0.1%–1%	Conventional electrospinning	Random, *D* = 600 ± 300–1000 ± 400 nm	Mechanical properties	Not specified	[138]
PU	DMAc	MWCNT (acid-treated)	3%	Conventional electrospinning	Random, *D* = 300–500 nm	Cell adhesion, proliferation, migration and aggregation	Not specified	[139]
PU	DMAc	MWCNT (acid-treated)	3%	Conventional electrospinning	Aligned, *D* = 300–500 nm	Cell proliferation, extracellular collagen secretion	Vascular	[140]
PVA/CS	AA/water (70 wt %)	MWCNT	0.99%	Electrospinning followed by crosslinking with GA vapor	Random , *D* = 157 ± 40 nm (non-crosslinked); 170 ± 43 nm (crosslinked)	Cell proliferation; protein adsorption capability	Not specified	[148]
SF	Water	MWCNT (functionalized with SDBS)	0.25%–1.5%	Conventional electrospinning	Random, *D* = 3 μm	Mechanical properties	Not specified	[136]
SF	Formic acid	SWCNT	1%	Co-electrospinning plus treatment with methanol and/or stretching	Random , *D* = 153 ± 99 nm Aligned, *D* = 147 ± 41 nm	Mechanical and electrical properties	Bone	[135]
SEBS	Toluene/THF (1:1)	MWCNT	1.5%	Conventional electrospinning	Random, *D* = 12.3 ± 3.6 μm Aligned, *D* = 10.2 ± 2.7 μm	Mechanical hysteresis and electrical conductivity	Not specified	[144]

N/A: Data not available; *D*: Diamater; The other acronyms are available in the acronym list.

**Table 3:** Examples of polymer-graphene electrospun scaffolds for tissue engineering.

Polymers (and additives)	Solvents	Nanocarbons	Filler loading (wt %)	Structure	Main improvements	Target tissue	Refs
CS/GEL/HA	AA/H_2_O	GO; RGO	2%	Random	Bioactivity, antibacterial and mechanical properties	Bone	[155]
CS/PEO/BC	AA/H_2_O	GO	0–2	Random *D* = 145–254 nm	Mechanical properties	Skin	[153]
CS/PVP/PEO	AA/H_2_O	GO	0–2	Random, *D* = 80–200 nm	Mechanical properties, bioactivity	Skin/bone	[152]
GEL	DMSO	GO-*g*-[P(HEMA-*g*-CL)]	2–3	Random, *D* = 100–200 nm	Mechanical and electrical properties, wettability	Not specified	[151]
PAN	DMF	GO; RGO	N/A	Random	Mechanical, electrical properties	Not specified	[157]
PCL	CHCl_3_	GO	N/A	Random, *D* = N/A	Mechanical, electrical, cell signaling	Skeletal muscle	[158]
PCL	CHCl_3_	GO	0.3–2	Random, *D* = 0.1–8 μm	Mechanical, electrical properties, bioactivity	Muscle	[159]
PCL	DMF	GO	0.3–0.5	Random; *D* = 1–3 μm	Cell differentiation	Nerve/cartilage	[8]
PCL	DMF	GO	0.5–2	Random, *D* = 0.2–2.5 μm	Mechanical properties, bioactivity, biodegradability	Bone	[160]
PCL	DCM/EtOH 4:1	GO; GO-*g*-PEG	0.25–2	Random, *D* = 200–1000 nm	Mechanical, wettability, cell adhesion	Osteochondral	[7]
PCL	AA	GO; RGO	0–1	Aligned, *D* = 100–400 nm	Mechanical properties	Not specified	[161]
PLA	CHCl_3_/DMF	GO; GO-*g*-PEG	2	Random, *D* = 500–1000 nm	Mechanical properties	Osteochondral	[28]
PLA/HA	DCM/DMF	GO	1–3	*D* = 412–516 nm	Mechanical, bioactivity	Bone	[162]
PLA/PU 4:1	DMF/DCM 2:3	GO	5	Random, *D* ~ 1 μm	Biocompatibility, antimicrobial properties	Cartilage	[163]
PLGA	THF/DMF	GO	1	*D* = 783–1461 nm	Wettability, bioactivity	Bone	[164]
PLGA/Col	HFIP	GO	4	Random, *D* = 100–950 nm	Cell proliferation, mechanical properties	Bone/muscle	[124]
PLGA/RGD	HFIP	GO	N/A	Random, *D* = 200–1440 nm	myogenic differentiation	Bone/muscle	[123]
PLGA/SF	HFIP	GO	1	Random, *D* = 130–280 nm	Mechanical, wettability, cell differentiation	Bone	[165]
PLLA	HFIP	GO	N/A	Aligned; *D* = 680 nm	Cell differentiation and growth	Nerve	[166]
PU	DMF	GO	0.5-2	*D* = 290–400 nm	Mechanical properties, bioactivity	Osteochondral	[167]
PVA	H_2_O	GNS	1%–7%	Random, *D* = 200-800 nm	Electrical properties	Cartilage	[168]
PVA	H_2_O	GO	0-5	Random, *D* < 1 μm	Mechanical properties, bioactivity	Bone	[169]
PVA/CS	AA/H_2_O	GO	0.05–0.6	*D* = 123–200 nm	Mechanical properties	Skin	[156]
PVC; FN	THF/DMF (4:1)	GO; RGO	N/A	N/A	Mechanical, electrical properties, bioactivity	Nerve	[170]
SF	H_2_O	GO; RGO	N/A	*D* = 3.9–5.2 μm	Electrical properties	Nerve	[154]

N/A: Data not available; *D*: Diamater; The other acronyms are available in the acronym list.

## Abbreviations

AA	Acetic acid
BC	Bacterial cellulose
CA	Cellulose acetate
CCM	Charge carrier mobility
CNT	Carbon nanotubes
Col	Collagen
CS	Chitosan
DCM	Dichloromethane
DMAc	*N*,*N*-dimethylacetamide
DMF	*N*,*N*-dimethyl-formamide
DMFA	Dimethylformaldehyde
DMSO	Dimethylsulfoxide
DWCNT	Double-walled carbon nanotube
E	Elastic modulus
ECM	Extracellular matrix
EtOH	Ethanol
FN	Fibronectin
GA	Glutaraldehyde
GEL	Gelatin
GNS	Graphene nanosheets
GO	Graphene oxide
GO-*g*-[P(HEMA-*g*-CL)]	Graphene oxide functionalized with poly(2-hydroxyethyl methacrylate)-graft-poly(e-caprolactone)
GO-*g*-PEG	Pegylated graphene oxide
HA	Hydroxyapatite
HFIP	1,1,1,3,3,3-hexafluoro-2-propanol
MWCNT	Multiwalled carbon nanotube
ND	Nanodiamond
NP	Nanoparticle
PAA	Polyacrylic acid
PAN	Polyacrylonitrile
PANI	Polyaniline
PBAT	Poly(butylene adipate-*co*-terephthalate
PCL	Polycaprolactone
PELA	Poly(ethylene glycol)-poly(d,l-lactide) copolymer
PEO	Polyethylene oxide
PGA	Polyglicolide
PhOMe	4-methoxyphenyl
PLA	Polylactic acid
PLCL	Poly(l-lactic acid-*co*-3-caprolactone)
PLGA	Poly(lactic-*co*-glycolic acid)
PLLA	Poly-l-lactide
PNIPAm	Poly(*N*-isopropyl acrylamide)
PNIPAm-*co*-MAA	Poly(*N*-isopropyl acrylamide-*co*-methacrylic acid
PU	Polyurethane
PVA	Poly(vinyl alcohol)
PVC	Polyvinylchloride
PVP	Polyvinyl pyrrolidone
RGD	Arginine-glycine-aspartic acid peptide
RGO	Reduced graphene oxide
SBF	Simulated body fluid
SDBS	Sodium dodecyl benzene sulfonate
SEBS	Styrene–ethylene/butylene–styrene
SF	Silk fibroin
SWCNT	Single-walled carbon nanotube
TE	Tissue engineering
THF	Tetrahydrofuran
TS	Tensile strength
